# Clinical Audit of the Use, Prescribing and Review of Anxiolytics and Hypnotics Within Acute Psychiatry

**DOI:** 10.1192/bjo.2026.11723

**Published:** 2026-06-30

**Authors:** Joseph Eremenko-Lowery, Suesan Rahmatalla

**Affiliations:** Tees, Esk and Wear Valleys NHS Foundation Trust, Darlington, United Kingdom

## Abstract

**Aims:**

To identify the use and prescribing of anxiolytics and hypnotics pre-admission, during the inpatient admission and on discharge and if such medications are reviewed during the inpatient admission on the adult mental health male and female wards.

**Methods:**

A list of patients admitted from 01-Dec-2024-28-Feb-2025 were collated using IBM Cognost Analytics. The electronic patient records (EPR) were used to identify patients who were discharged after 28-Feb2-2025, and such patients were excluded from the audit. Medications prescribed pre-admission were identified using the medicines reconciliation in the EPR. Medications prescribed during the inpatient admission and on discharge were found on the electronic prescribing system and EPR. Microsoft forms were used to collate data and Microsoft excel was used for data analysis. Lorazepam, promethazine, diazepam and zopiclone prescriptions were analysed.

**Results:**

A total of 100 prescriptions on the female and 35 prescriptions on the male were prescribed during the inpatient admission. 26% (female) and 46% (male) of prescriptions were reviewed throughout the inpatient admission and documented. 6% (female) and 0% (male) of prescriptions were reviewed weekly. 9% (female) and 14% (male) prescriptions were changed in terms of dose, frequency and/or maximum dose.

A total of 48 (female) and 18 (male) prescriptions on the male wards were prescribed upon discharge. 71% (female) and 22% (male) prescriptions were changed in terms of dose, frequency and/or maximum dose. 74% (female) and 91% (male) of discharges had a documented plan regarding the review of benzodiazepines and/or z drugs.

**Conclusion:**

To conclude, the audit demonstrates poor compliance of the TEWV trust policy regarding regular reviews of anxiolytics and z-drugs and documentation during the inpatient admission. The female ward had a greater number of patients and prescriptions, which was attributed to the high turnover of patients. However, on discharge there was a higher percentage of prescriptions that were reviewed, changed and documented. Evidenting that majority of prescriptions were reviewed at the point of discharge. This poses a risk to patient safety especially when discharged to a least restrictive environment in which symptoms of withdrawal cannot be closely monitored when compared to an inpatient setting. This audit encourages clinicians to ensure regular reviews are maintained through weekly reviews with the MDT and patient, and more training to staff, particularly doctors in training.

